# Sublethal Injury and Viable but Non-culturable (VBNC) State in Microorganisms During Preservation of Food and Biological Materials by Non-thermal Processes

**DOI:** 10.3389/fmicb.2018.02773

**Published:** 2018-11-20

**Authors:** Felix Schottroff, Antje Fröhling, Marija Zunabovic-Pichler, Anna Krottenthaler, Oliver Schlüter, Henry Jäger

**Affiliations:** ^1^Institute of Food Technology, University of Natural Resources and Life Sciences, Vienna, Austria; ^2^Quality and Safety of Food and Feed, Leibniz Institute for Agricultural Engineering and Bioeconomy, Potsdam, Germany; ^3^Institute of Food Science, University of Natural Resources and Life Sciences, Vienna, Austria

**Keywords:** viable but non-culturable (VBNC), sublethal injury, high hydrostatic pressure (HHP), pulsed electric fields (PEFs), pulsed light (PL), ultraviolet (UV) radiation, cold plasma (CP), flow cytometry

## Abstract

The viable but non-culturable (VBNC) state, as well as sublethal injury of microorganisms pose a distinct threat to food safety, as the use of traditional, culture-based microbiological analyses might lead to an underestimation or a misinterpretation of the product’s microbial status and recovery phenomena of microorganisms may occur. For thermal treatments, a large amount of data and experience is available and processes are designed accordingly. In case of innovative inactivation treatments, however, there are still several open points with relevance for the investigation of inactivation mechanisms as well as for the application and validation of the preservation processes. Thus, this paper presents a comprehensive compilation of non-thermal preservation technologies, i.e., high hydrostatic pressure (HHP), pulsed electric fields (PEFs), pulsed light (PL), and ultraviolet (UV) radiation, as well as cold plasma (CP) treatments. The basic technological principles and the cellular and molecular mechanisms of action are described. Based on this, appropriate analytical methods are outlined, i.e., direct viable count, staining, and molecular biological methods, in order to enable the differentiation between viable and dead cells, as well as the possible occurrence of an intermediate state. Finally, further research needs are outlined.

## Introduction

Depending environmental conditions and stresses, microorganisms on exist in different metabolic states and growth phases whereas active replication of cells is not included in all the states (Davis, 2014). In this context, viability is commonly referred to as the existence of replication in culture media (Espina et al., 2016) and the cultivation based viability assay is still considered the “gold standard” for the determination of bacterial viability, even though the absence of growth is not a clear indicator for the absence of microbial life (Emerson et al., 2017). The definition of life or dead is difficult for microorganisms because the route from life to death as well as the reverse way of recovery is still uncertain and includes many different states. The growth or the lack of growth on or in culture medium allows different interpretation of the results. The formation of colonies on growth medium means that at least one viable cell was able to replicate. Additionally, it is possible that more than one viable cell concur at the same place and form only one colony which might lead to an underestimation of viable cells. The lack of colony growth on culture medium implies that no viable cell is in the sample. However, another interpretation is the use of incorrect growth medium and conditions, i.e., temperature and time, or damaged or stressed cells that are not able to form colonies. Nevertheless, these cells can be viable (Davey, 2011). This again might lead to an underestimation of viable cells. The loss of culturability of microorganisms can be the consequence of damages to essential cellular components or the lack of essential cellular components. This damage can be of a temporary, i.e., sublethal damage, or permanent nature, i.e., lethal injury (Kell et al., 1998). Since there is often an improper classification of injured cells as VBNC cells (Pinto et al., 2015), a description of the different metabolic states of microorganisms is given here. The formation of bacterial endospores is a well-known long-term survival mechanism of Gram-positive bacteria (Colwell, 2009). Dormant bacteria are in a physiological state characterized by a shutdown of the metabolism. These cells show neglectable metabolic activity that cannot be detected by vital assays and they might not be culturable. Upon specific stimuli these cells regain activity and can thus be cultivated again (Kell et al., 1998). Persistent cells are defined as an antibiotic-resistant subpopulation, whereas the other cells in the population remain sensitive to the antibiotics (Li et al., 2014). Low numbers of these cells are formed during growth (Pinto et al., 2015). The viable but non-culturable (VBNC) state of cells can be defined as inactive form of life that is induced by stressful conditions (Colwell, 2009) and undergoes recovery under suitable conditions (Ramamurthy et al., 2014). VBNC cells show low but detectable metabolic activity, they maintain membrane integrity, and express genes at low levels but the formation of colony forming units (CFUs) on culture media is inhibited (Ayrapetyan and Oliver, 2016). The process of recovery of VBNC cells is called resuscitation and describes the transition from the non-cultural to the cultural state (Kell et al., 1998) without any change in cell number due to regrowth (Bogosian and Bourneuf, 2001). Resuscitation can be triggered by the increase of nutrient concentration, decrease or increase of the temperature, addition of chemical stimuli, and co-cultivation with host cells. However, the greatest challenge is to distinguish real resuscitation from regrowth of residual culturable cells (Zhao et al., 2017). The formation of VBNC cells occurs upon longer periods of incubation under stressful conditions (Pinto et al., 2015). In contrast to VBNC cells, sublethally injured species still possess the ability to grow, however, solely and predominantly on non-selective growth media (Li et al., 2014). Sublethal injury in bacteria is induced by the exposure to chemical or physical processes. Reversible damage of cell structures and loss of cell functions can be the result of a sublethal treatment (Wesche et al., 2009). These injured cells usually possess the ability to repair their damages under suitable conditions and consequently they are able to grow again (Espina et al., 2016). Severely injured cells that cannot be resuscitated under laboratory conditions may enter the VBNC state and maintain their pathogenicity (Wesche et al., 2009).

It is well known that less severe preservation intensities result in non-injured, injured, and inactivated bacterial populations (Silva et al., 2012; Wu, 2014; Schottroff et al., 2017), and the VBNC state can also be induced by preservation treatments (Oliver, 2010; Ayrapetyan and Oliver, 2016). Thus, the detection of injured and VBNC bacteria is of distinct relevance for industrial food production, to avoid false-positive or false-negative results (Wu, 2014). Especially an under-estimation of the product’s safety status might lead to severe consequences, as the presence and potential re-growth of microorganisms in a physiologically infringed state might lead to a reduction of the shelf life or to an outbreak of foodborne illnesses, and thus may represent a potential risk for food safety (Wesche et al., 2009; Silva et al., 2012). Therefore, reliable detection methods for sublethally damaged cells and VBNC cells are necessary to study inactivation mechanisms, to assess the effectiveness of preservation treatments, to provide suitable validation concepts and to ensure food safety.

Thus, the present paper gives an overview of non-thermal decontamination technologies and their principles of action. The occurrence of sublethal injury and the VBNC state as induced by the treatments are discussed. Consequently, a set of methodologies to differentiate the individual physiological states is presented.

## Overview of Non-Thermal Inactivation Technologies

Food preservation techniques should fulfill special requirements, including prolonged shelf life of perishable products and maintenance of the individual safety status, by inactivation of pathogenic bacteria and spoilage microorganisms. Additionally, organoleptic and nutritional properties of the products should not be changed and the formation of process induced contaminants should be avoided (Birlouez-Aragon et al., 2010). Last but not least, the preservation technique should be cheap and convenient to apply and there should be no concerns from legislation and consumers (Raso and Barbosa-Cánovas, 2003). An increasing focus on minimal processing with the aim to maintain or improve food safety while lowering the negative impact of processing on product quality leads to the growing implementation of non-thermal preservation technologies. Thus, in the following paragraph promising non-thermal preservation technologies will be summarized and their principles of action will be explained. More detailed insights into application concepts for different food and biological products are given by Knorr et al. (2011).

### High Hydrostatic Pressure

High hydrostatic pressure treatment (HHP) is the most established non-thermal technology in industrial food production and capable of the successful reduction of vegetative pathogenic and spoilage bacteria, with a minimal degradation of valuable food constituents (Gayan et al., 2017).

#### Principle of the Technology

Current applications of HHP are solely operated in batch mode, with the treatment of the final product being carried out within a flexible packaging. For this purpose, the packaged goods are placed in a pressure vessel, which is linked to a high pressure pump and a corresponding pressure intensifier (Elamin et al., 2015). The vessel is filled with a pressure transmitting liquid, e.g., water, and additional water is pumped into the system in order to increase the pressure. During the pressure built-up, the so-called adiabatic heating occurs, i.e., a product-dependent temperature increase caused by compression (Ragstogi, 2013). Thus, HHP units are further equipped with temperature sensors, in order to be able to monitor this adiabatic heating. HHP treatment is a so-called isocratic process, i.e., the pressure transmission is immediate and its distribution is multidirectionally homogeneous. Consequently the same pressure treatment applies to all goods within the treatment chamber, regardless of product size and shape (Elamin et al., 2015). However, temperature non-uniformity may occur during the treatment due to heat losses toward the wall of the pressure vessel. After the dwell time, the pressure is released and the product can be further handled. Industrial processes are typically carried out in a pressure range of 200–600 MPa, with holding times of up to 10 min with typical temperature increase rates of around 3 to 9°C per 100 MPa, thus allowing pasteurization at distinctly lower temperatures compared to thermal processes (Ting, 2010; Knorr et al., 2011; Ragstogi, 2013).

#### Inactivation and Mechanisms

In general, the effect of HHP on microorganisms is based on the principle of Le Châtelier and Braun, i.e., the external force applied on a product in the form of an increased pressure causes a shift of the thermodynamic equilibrium in such a way that (biochemical) molecules are reduced in volume (Gayan et al., 2017). Furthermore, the principle of microscopic ordering states that under HHP conditions, the degree of ordering of molecules is increased, further changing the thermodynamic behavior of molecules, such as melting temperature (Balny and Masson, 1993).

**FIGURE 1:**
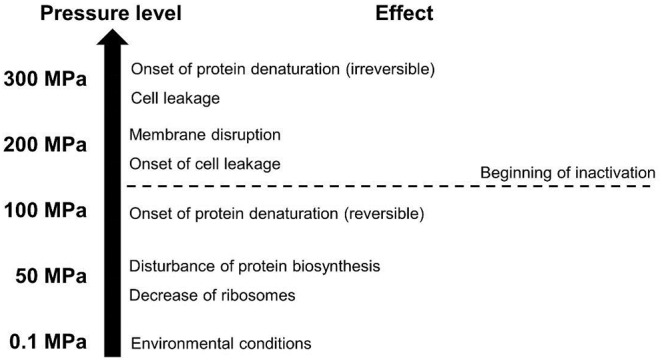
General effects of different pressure levels on microbial cells (adapted from Lado and Yousef, 2002).

Vegetative forms of microorganisms typically present in food, especially pathogenic and spoilage organisms, are the target of the HHP treatment, as they can effectively be inactivated at ambient temperature (Mota et al., 2013; Georget et al., 2015; Wang et al., 2016). The inactivation effectiveness of the HHP process on vegetative microorganisms is based on a multitude of different factors affecting cell components (see Figure 1). Thus, pressure acts on morphology and internal structures of cells but also on the metabolism (Bartlett, 2002; Mota et al., 2013). Primarily, membranes, ribosomes, as well as proteins and enzymes are affected.

The effect of HHP on membranes is based on pressure-induced phase transitions and alterations in the membrane fluidity, causing membrane disintegration as well as denaturation of membrane-associated proteins and the associated decrease of the membrane’s barrier function (Winter and Jeworrek, 2009). Furthermore, membrane disruption also seems to be associated with the emergence of reactive oxygen species (ROS) and an accompanying oxidative stress, leading to a further inactivation in *Escherichia coli* (Aertsen et al., 2005; Kimura et al., 2017).

Moreover, ribosomes are disrupted by pressure, thus disturbing protein biosynthesis and the associated intracellular metabolism (Niven et al., 1999). The main target of the pressure, however, is the denaturation of proteins and enzymes. This is caused by weakening of non-covalent chemical bonds, such as electrostatic or hydrophobic interactions. Thus, the three-dimensional tertiary and quaternary structures of proteins are altered under pressure, in order to reduce the total volume (Silva, 1993; Winter and Dzwolak, 2005). Above a pressure level of 400 MPa protein monomers are denatured, resulting in the so-called molten globule configuration (Roche et al., 2012; Gayan et al., 2017).

Another distinct influence factor on the inactivation of microorganisms during HHP treatment is the change of intracellular pH due to the shift of the dissociation equilibrium under pressure, which is also related to the pressure-induced denaturation of pH-buffering enzymes and membrane disruption (Molina-Gutierrez et al., 2002). As a stable pH within the cells is inevitable for the maintenance of an intact metabolism and viability, this pH shift also contributes to microbial death under high pressure (Knorr et al., 2011).

Due to genetic as well as phenotypical variations among different forms of microbial life, variations in pressure resistance are present. Thus, eukaryotic species are usually less resistant than prokaryotes, with Gram-negative bacteria being less resistant than Gram-positive ones (Considine et al., 2008; Dumay et al., 2010; Georget et al., 2015).

The effectiveness of the treatment can be increased by the use of slightly elevated process temperatures (Knorr et al., 2011), and especially for sterilization applications the use of higher temperatures (90–100°C) is necessary to achieve a sufficient level of inactivation (Wimalaratne and Farid, 2008; Sevenich et al., 2014; Sevenich and Mathys, 2018).

Inactivation kinetics of high pressure treated microorganisms typically exhibit a so-called tailing behavior, i.e., a decrease in inactivation levels compared to a linear progression, toward the end of the treatment, with increasing process intensities. Reasons for this tailing might be due to the occurrence of microbial subpopulations with different individual pressure resistances due to genetic diversity, as well as adaption to external stresses and selection (Mota et al., 2013).

#### Role of Sublethal Injury and the VBNC State

Pressurized microorganisms react to the external force by the execution of different stress responses, leading to potential changes in physiology, and associated adaption as well as an increased resistance against the treatment (Mota et al., 2013). Depending on the pressure level and dwell time, as well as intrinsic microbial factors, repair mechanisms might lead to recovery subsequent to the treatment, i.e., pressure-induced injuries may be reversed, with the consequence of regrowth (Bozoglu et al., 2004).

Several studies demonstrated the recovery of microorganisms subsequent to a HHP inactivation treatment, indicating sublethal damage caused by the process and the associated repair of the damage.

Bozoglu et al. (2004) evaluated the influence of temperature on the recovery of *Listeria*, *Staphylococcus*, *Escherichia*, and *Salmonella* species after HHP treatment (maximum 550 MPa, 10 min) in milk and found that at 4°C only *Listeria monocytogenes* was able to regain viability, whereas at 22 and 30°C also colonies of the other cultures could be detected. Similar results were obtained by Bull et al. (2005) for *Listeria monocytogenes*, emphasizing the distinct temperature dependence of recovery after HHP treatment and the associated implications for food safety. Ritz et al. (2006) treated *L. monocytogenes* and *Salmonella* Typhimurium in buffers at pH 7 and 5.6, with pressures of 350–600 MPa for 10 min. They evaluated the behavior of the seemingly inactivated cultures during storage at 4 and 20°C and found resuscitation for all trials below 600 MPa, with a higher amount of viable populations present at increased storage temperature.

Ananta et al. (2004) processed *Lactobacillus rhamnosus* in PBS buffer in the range of 100–600 MPa. Using flow cytometry and propidium iodide as well as cFDA as dyes (see the Section “Detection of Physiological and Structural Changes”), it could be shown that although cultivation on agar plates was not possible, the cell’s metabolism (esterase activity) was not in all cases completely shut down. Also in the process window of a lethal treatment, cases were presented where the bacterial membrane was still (partially) intact. Kilimann et al. (2005) showed that upon a HHP treatment of *E. coli* in the range of 200–400 MPa pH homoeostasis was permanently disturbed. On the other hand, the recovery of the cells subsequent to the treatment was distinctly increased when glutamate, which is known to stabilize the intracellular pH, was added to the medium at acidic conditions (pH 4). Kimura et al. (2017) investigated the high pressure inactivation of *E. coli* cells in the range of 400–600 MPa and analyzed viability using flow cytometry as well as culturability of the cells subsequent to the treatment. They found that sublethal injury and recovery was present for the treatments, especially in the lower range of the investigated pressure levels, as distinctly higher CFUs were present after incubation when oxygen-scavenging pyruvate was added to the agar plates. Further, growth temperature after the HHP treatment influenced the recovery of the cells, as higher counts were detected at 25°C incubated cultures, in comparison to 37°C. Also, growth of pressure injured cells was associated to a distinct lag phase at 25°C, indicating an adaption to the growth conditions and the onset of repair mechanisms. In general, specific product properties can exert pronounced effects on the recovery of microorganisms, especially in terms of osmoregulation. Thus, high sugar or salt concentrations in the matrix can decrease the susceptibility of the cells toward high pressure, associated with the accumulation of compatible solutes inside the cell (Molina-Hoppner et al., 2004; Gayan et al., 2017).

Besides, pressure resistance acquisition in bacteria is possible, when subpopulations that survived HHP treatment are re-grown and treated again. This resistance formation was described for a variety of microorganisms with implications for food safety and product quality, involving enterobacteriaceae, *L. monocytogenes, S. cerevisiae*, and *Lactobacillus* spp. (Mota et al., 2013).

Furthermore, Bozoglu et al. (2004) differentiated between two states of injury, one less severe type being associated to the cell envelope, and a more pronounced damage, linked to a negative interference of metabolic processes, similar to the observations made for sublethally injured cells by PEF treatment (Jaeger et al., 2009), with the latter state being associated with the VBNC state (no growth on non-selective agars).

As inactivation kinetics of HHP treated microorganisms often exert a so-called tailing, indicating the presence of resistant subpopulations, as well as the possibility of resuscitation and the associated regaining of viability, it has to be ensured that preservation processes involving high pressure are designed in such a way that they are severe enough to completely inactivate the bacterial target populations or to design processing concepts for the specific control and avoidance of recovery, based on the hurdle concept. Thus, traditional culturability tests might not be sufficient to assess the status of the microorganisms after the treatment. Consequently, adequate analytical methods (see the Section “Detection of Physiological and Structural Changes”) have to be carried out, in order to obtain reliable data considering the microbial status of a decontaminated product.

### Pulsed Electric Fields

Pulsed electric fields (PEFs) are used in a variety of different food processing applications, such as stress induction in microorganisms, mass transfer enhancement for food products or shelf life extension by microbial inactivation. Especially for the latter purpose, this technology bears a great potential, as it enables the efficient inactivation of vegetative microorganisms at distinctly lower process temperatures, in contrast to conventional heat treatments (Schottroff et al., 2017).

#### Principle of the Technology

In general, PEFs are generated by charging of a capacitor bank and controlled discharging, e.g., via spark gaps or semiconductor switches. Depending on the type of switch, the pulse shape can either be exponentially declining or rectangular. The applied voltage is usually in the kilovolt range, with electric field strengths (ratio of voltage and electrode distance) between 10 and 50 kV cm^-1^ for microbial inactivation purposes. Furthermore, monopolar or bipolar modes of operation can be realized, with the latter being beneficial for the reduction of electrode corrosion (Barsotti et al., 1999). The electric field exposure of the product is realized within a treatment chamber, consisting of at least two electrodes, usually from stainless steel, which are separated by a non-conductive insulator. The most widespread treatment chamber configurations are parallel plate for batch applications and the co-linear configuration for continuous processes (Huang and Wang, 2009; Toepfl, 2011).

The duration of one electric pulse is usually in the range of several microseconds (μs) to a few milliseconds (ms) (Heinz et al., 2002).In order to achieve an effective inactivation and the targeted log reduction in microbial counts, a number of pulses are applied, and the total specific energy input (W_spec_) is usually used as the criterion for the required process intensity. This cumulative parameter condenses the values of electrical and process parameters, and comprises voltage (U), current (I), pulse duration (τ), frequency (f) as well as the product mass (m) or mass flow (ṁ). Typical values of W_spec_ are in the range of 40–200 kJ kg^-1^, with a corresponding ΔT of up to 45 K (Toepfl, 2006; Amiali and Ngadi, 2012). As the specific energy input is directly linked to temperature increase, this value is often used to control and monitor the process (Witt et al., 2017). An overview of relevant process variables to be considered during a PEF treatment are given by Raso et al. (2016).

#### Inactivation and Mechanisms

**FIGURE 2:**
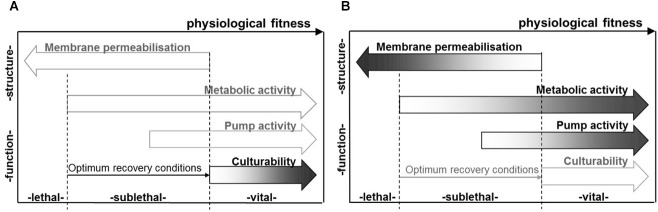
Factors relating to the physiological state of microbial populations subsequent to PEF treatment, as determinable based on cultural methods, e.g., using selective media **(A)**, as well as membrane integrity, metabolic and pump activity, as indicated, e.g., by staining procedures **(B)**. Reprinted by permission from Miklavčič (2017).

The inactivation mechanism during PEF treatment is based on electroporation and can be considered non-thermal (Knorr et al., 2011). However, an energy-dependent increase in process temperature is present during PEF treatment, due to electric current flow and the individual resistance of the product and may contribute to additional inactivation effects. The technology can be used for the shelf life extension of heat sensitive products, predominantly liquid foods (Lasekan et al., 2017) as an alternative to the conventional thermal pasteurization. An overview of different physiological states of microbial cells after PEF treatment and selected measures for their evaluation are given in Figure 2.

In order for non-thermal inactivation effects to occur, the electric field strength has to exceed a certain, microorganism-dependent value, usually in the range of 10–15 kV cm^-1^ for bacteria and around 5 kV cm^-1^ for yeasts (Grahl and Märkl, 1996). Above this critical value, the occurring electro-compressive forces across the membrane, which are emerging due to electric field-induced accumulation and attraction of oppositely charged ions on the inside and outside of the microbial cell wall – are strong enough to cause a perforation of the membrane (Neumann and Rosenheck, 1972). Depending on the intensity of the electric field, the electroporation effect can either be reversible, i.e., the pores can be sealed again, or irreversible, with an associated permanent loss of the membrane’s barrier function leading to death of the microbial cell (Zimmermann et al., 1974; Kinosita and Tsong, 1977).

The onset of non-thermal effects during PEF treatment strongly depends on the size of the microorganism to be treated, with larger cells being distinctly easier to inactivate than smaller cells, due to a higher surface area exposed to the electric field (Schwan, 1957; Heinz et al., 2002). Furthermore, also the Gram behavior of a bacterium influences its resistance against the PEF treatment. In general, Gram-negative organisms are more sensitive to the treatment than Gram-positive species – due to the alteration of the membrane’s electrical properties by the peptidoglycan layer – and can therefore be inactivated more easily (Hulsheger et al., 1983). However, more precise studies have shown a pH dependence of this resistance and pH can also be considered as one of the major matrix variables to affect the sensitivity of microbial cells toward PEF inactivation.

García et al. (2005b) compared the inactivation behavior five different Gram-positive and five different Gram-negative strains and found that resistance against PEF treatment was greatest at pH 7 for Gram-positive bacteria, whereas Gram-negatives were more resistant at pH 4.

Moreover, not only microbiological factors influence the inactivation but also further properties of the medium the microorganisms are suspended in. As the electrical conductivity influences the current flow through a medium, this strongly influences the energy to be delivered by each electrical pulse (Grahl and Märkl, 1996). Thus, at a constant voltage and pulse width, the energy per pulse decreases with a decreasing conductivity, due to a decreasing current. In order to obtain a constant total specific energy input, the frequency would have to be increased or the mass flow would have to be decreased (Schottroff et al., 2017). Moreover, the pH of the treated product may exert strong effects on the inactivation efficiency by PEF. During PEF treatment of microorganisms in a low pH medium, the molecules of the acid might diffuse through the electrically induced pores into the cytoplasm, with the consequence of a lowered intracellular pH and the associated disturbance of the cell’s metabolism. Similar effects can be observed when antimicrobials are present within the treated matrix (Barbosa-Canovas et al., 2000; Garcia et al., 2007).

Considering bacterial endospores, however, the electric field alone is not effective for inactivation purposes, due to the lack of a distinct cytoplasmic membrane in spores. On the other hand, it could be shown that in combination with elevated temperatures PEF may lead to a more pronounced inactivation than heat alone (Siemer et al., 2014; Reineke et al., 2015). However, further research is necessary in this field in order to obtain mechanistic insights and a broader database on the effects of PEF assisted thermal sterilization on different spore species in various matrices.

#### Role of Sublethal Injury and the VBNC State

As the electric field mainly affects the cell wall and membrane of microorganisms (Pillet et al., 2016), especially in case of reversible electroporation it is possible that the damage of the membrane is not severe enough to cause an inactivation of a microbial cell. Thus, under certain circumstances, e.g., presence of nutrients, suitable water activity, ideal growth temperature and pH, the microorganism might be able to reseal the membrane pores and consequently regain viability (Garcia et al., 2007). Thus, subsequent to PEF treatment, three different states of a microbial population might occur, i.e., alive, dead, and sublethally injured subpopulations, with sublethal injury referring to a physiological state in-between alive and dead (Wang et al., 2018). Depending on microbial and media factors as well as treatment intensity, only one of these subpopulations might be present, but also several subpopulations can co-exist. During storage, the status of some of the individual populations can also merge into each other (Schottroff et al., 2017). From a mechanistic point of view, a loss of the cell’s metabolic activity as well as the membrane’s barrier properties refers to the inactivated, e.g., dead, subpopulation, whereas an active state of these two microbial constituents refers to the living state. If the membrane is perforated but the metabolic activity is still active, the sublethal condition is present. In this case, the injured population can subsequently develop into a dead or alive subpopulation (Ulmer et al., 2002; Jaeger et al., 2009; Schottroff et al., 2017). The occurrence of sublethal injury depends on a variety of different factory, including product properties, microbial species, and treatment severity (García et al., 2003, 2005a; Zhao et al., 2011). García et al. (2005b) showed a pH-dependent emergence of sublethal injuries, with the greatest amount of sublethally injured cells being associated to the pH level at which the greatest resistance to the treatment was determined. On the other hand, no sublethal subpopulations were present at pH levels with a low resistance against the treatment. Subpopulations being able to overcome the sublethal state are usually not fully harmed and are thus able to recover and repair the damage caused by the PEF treatment, i.e., repair of the membrane but also of metabolic disturbances (Garcia et al., 2007). Due to individual resistances within a certain microbiological population, resealing of PEF-induced membrane damage can also appear above the critical field strength, and at pH levels which would usually lead to an inactivation of the cell (Sagarzazu et al., 2013). As shocks from an electric field are a phenomenon which is not present in nature, it triggers a variety of different stress responses within the microbial cell. Thus, also the recovery process involves a distinct range of individually regulated genes. An overview of stress responses and repair mechanisms subsequent to PEF treatment is given by Schottroff et al. (2017).

Furthermore, some microorganisms are able to distinctly enter the VBNC state subsequent to PEF treatment. Rowan (2004) describes the occurrence of this physiological state for PEF-treated *Bacillus cereus* and *Listeria monocytogenes*, whereas the same research group later showed that the VBNC state occurred after heat, but not after PEF treatment of *E. coli*, *Bacillus cereus*, and *Listeria monocytogenes* (Yaqub et al., 2004). Thus, further research on this issue is needed, also with regard to the significance of this physiological state for an improved food safety.

### Pulsed Light and Ultraviolet Radiation

Pulsed light (PL) is also known as pulsed UV-light (PUV), intense pulsed light (IPL), high-intensity pulsed UV light (HIPL), high-intensity broad-spectrum UV light (BSPL), intense light pulsed (ILP), and pulsed white light (PWL) (Heinrich et al., 2016b). The application of this decontamination technology has been well demonstrated in the packaging industry. Also, the application of ultra violet (UV) could successfully be implemented for food and packaging surface decontamination, as it is effective against a great variety of pathogens (including bacterial endospores) and spoilage microorganisms (Van Impe et al., 2018).

#### Principle of the Technology

After several adaptions and patents of the PL technology, the FDA approved the application “in the production, processing and handling of food” (Food and Drug Administration, 1996). PL can be applied in several processing steps in the food chain (Heinrich et al., 2016b). In principle, during PL treatment, short-duration, high-power electromagnetic pulses are emitted from a specific flash lamp filled with inert gas. Different lamp types are available in various shapes and materials almost exclusively filled with xenon gas and partly with other noble gases (Dunn et al., 1989; Gómez-López et al., 2007). Considering the application of xenon-flash lamps the emitted broad-spectrum radiation ranges from 180 to 1,100 nm, which encompasses infrared (700–1,100 nm), visible light (400–700 nm) and a fraction of ultraviolet light (180–400 nm) (Dunn, 1996). The lamps are positioned above, below or surrounding the target object in a tightly closed treatment chamber or tunnel. The components of the basic equipment comprise (i) a power unit for generation of high-power pulses (ii) treatment chamber where pulses are transformed into high-power light pulses (Heinrich et al., 2016b).

Pulsed light is described as cost-effective, non-thermal decontamination technology without unwanted residuals on foods. It has been further developed from the conventional continuous-wave (CW) UV light of defined wavelength. It has been demonstrated that PL is more effective than CW-UV due to its high peak power (Kramer et al., 2016). Nevertheless, a specific UV range is proven to be more effective and varies with the target organism (Kramer et al., 2016). The mode of inactivation between the two technologies is still under discussion.

The principle parameters to describe the effect of PL is the fluence rate (F) [W m^-2^], the fluence (F) [J m^-2^], the number of pulses (*n*), pulse width (*t*) [s], exposure time (*t*_tot_ = *n*^∗^*t*) [s], frequency [Hz], and the peak power [W] (Heinrich et al., 2016b). Fluence is photochemically seen the most appropriate parameter to describe PL efficacy as it allows to measure the amount of energy impinging the target object (Rowan et al., 2015). Hence, the microbial inactivation dynamics only depend on the fluence applied (Rowan et al., 2015).

Considering several publications on the applicability of PL on different food types and surfaces, basic information on the parameters use is lacking which makes research outcomes incomparable (e.g., lamp manufacturer, geometry of target matrix, inoculation trials, etc.). In food processing PL is mainly used for surface decontamination of food and non-food products. Decontamination efficacy increases when reflection is low, and absorption and transmission coefficients are increased (Gómez-López et al., 2007; Heinrich et al., 2016b). Hence, a surface without huge irregularities and light absorbing matter might protect the target microorganism from the light source. The application of colored agar or concentration dependent protein solutions hamper light penetration mainly at the UV-C range (Kramer et al., 2016).

#### Inactivation and Mechanisms

Pulsed light is able to inactivate a range of microorganisms in short processing times (seconds) on different matrices. Depending on the type of microorganism up to 6 log units of inactivation are reported whereas on rough surfaces, such as meat products, lower colony count reduction up to 3 log units were found (Heinrich et al., 2016b; Zunabovic et al., 2017; Van Impe et al., 2018).

The characterization of the inactivation kinetics has been properly demonstrated by several authors (Rowan et al., 2015; Heinrich et al., 2016a; Van Impe et al., 2018).

A “typical” PL inactivation curve is mostly postulated to be non-linear with sigmoid shape in three phases. Firstly, (i) cell injury at initial plateau or shoulder effect (ii) fast increase of the inactivation and finally (iii) the tailing phase due to several factors of survival and strain-specific effects (McDonald et al., 2000; Gómez-López et al., 2007; Farrell et al., 2010). Heinrich et al. (2016a) studied the inactivation kinetics on different *Listeria monocytogenes* strains in single application instead of strain cocktails. The substantial decline of cells with less pronounced shoulder was shown already after 0.46 J cm^-2^. The shoulder effect is absent in case of high initial fluence (Farrell et al., 2010). It must be emphasized here that the application of kinetic models requires particular combinations of conditions (e.g., amount of data points, minimum log reduction, etc.).

Several constituents of microorganisms are known to contribute to the susceptibility toward PL due to the current physiological state and density of the population, and further the growth rate and lag time (Dunn et al., 1989). Pigmentation of microorganisms (e.g., *Aspergillus* conidiospores, melanin, alginate slime, pyocyanin) exhibit higher resistance to PL (Kramer et al., 2016). In addition, a Gram-behavior dependent susceptibility of microorganisms to PL was shown in several studies, with a generally greater tolerance of Gram-positive bacteria, compared to Gram-negative species (Heinrich et al., 2016b; Kramer et al., 2016).

The inactivation mechanisms according to Van Impe et al. (2018) for PL/UV-based technologies can be summarized in a range from strong to lower relevance: (i) damage incl. oxidative damage to cell membrane and damage to DNA (ii) damage to spore coat and (iii) inactivation of key enzymes and chemical modification of in spore core incl. cortex.

The bactericidal effect of PL is contributed by the UV fraction (mainly UV-C), causing photochemical alterations of the genome by emergence of cyclobutane thymine dimers (CPD) and other DNA lesions (Goosen and Moolenaar, 2008; Rowan et al., 2015). At the level of RNA single stranded breaks and formation of dimers are observed (Pollock et al., 2017). Photophysical effects comprise cell death through irreversible structural damage of cells and photothermal mechanisms achieve cell death due to disruption and explosion (Dunn et al., 1989; Wekhof, 2000; Cheigh et al., 2012; Rowan et al., 2015). The type of effect/damage strongly depends on the microorganism and the experimental setup. These inter-related mechanisms act in parallel or in sequence. *Escherichia coli* for instance is inactivated at 270 nm due to high absorption of the DNA at this wavelength (Wang et al., 2005). In addition, the evolutionary adaption of some bacteria frequently exposed to sunlight might contribute to higher PL resistance. DNA repair repertoires (photolyase, glycosylase, endonuclease or nucleotide excision repair) reducing UV-induced lesions reduce susceptibility of certain bacterial strains. These repair pathways are thoroughly discussed in the review of Goosen and Moolenaar (2008). Mucoid and pigment forming bacteria at high cell densities showed increased PL resistance (e.g., *Pseudomonas aeruginosa* strains) (Farrell et al., 2010). Fungal and bacterial spores show different susceptibility after PL treatment due to absorbing spore colors (Dunn et al., 1991; Levy et al., 2012). The size of bacteria is another crucial factor for PL resistance, as larger cells are generally more susceptible to the treatment than smaller organisms (Wekhof, 2000).

Studies on the viral inactivation on food-related surfaces are scarce. The application of the technology in the drinking water sector is more prominent in scientific literature. Here, mainly poliovirus and rotavirus 4 to 10 log reduction in relation to water turbidity is studied (Vimont et al., 2015). Even though PL is described as non-thermal, product heating cannot be excluded after long operation times. This may additionally lead to microbial inactivation with regard to the target matrix.

#### Role of Sublethal Injury and the VBNC State

Kinetic data attributed to PL treatments reveal concrete differences due to processing conditions, initial contamination level, matrix effects, etc. Nevertheless, the underestimation of microbial survivors is evident as these kinetic models rely on growth-dependent methodologies (culturability). Limited molecular and cellular based studies are available on the examination of VBNC fractions after PL treatments. As expected, the differences between culture and non-culture based techniques are huge (Rowan et al., 2015). Immediate loss of bacterial vitality after PL application could not be shown even if a cultural 6 log destruction was examined (Rowan et al., 2015). Cell populations with elevated metabolic activity are more vulnerable against PL treatment than older cultures that obviously exhibit better repair mechanisms (Kramer et al., 2016). This has been shown for *E. coli* and *Candida* strains. Gómez-López et al. (2005) observed so-called photoreactivation after PL treatments. This recovery form of PL-treated microorganisms arises after exposure to visible-light. The so-called photolyase enzyme reverses the joinings of two adjacent pyrimidines under light in the near UV/blue light region. Photoreactivation was also shown for *L. innocua*, especially after immediate illumination *post* PL (Kramer et al., 2016). Photoreactivation has been demonstrated a time dependent mechanism, most effective within the first 30 min for some bacterial cultures (Kramer et al., 2016). Further details on the UV repair mechanisms are reviewed by Goosen and Moolenaar (2008). Cellular responses of *Candida* strains to PL treatments depend on the UV-dose applied. An increase of the cell membrane permeability correlated with specific patterns of ROS during treatments (Rowan et al., 2015). Sublethal PL exposure of yeast cells proved cellular repair. Flow cytometric examinations showed early loss of culturability of *S. cerevisiae* strains rather shutdown of vitality indicators (Ferrario et al., 2014). This could also be observed for Gram-positive and negative representative bacteria, such as *Listeria innocua* and *E. coli*. Despite the reduction of colony counts below the detection limit at fluences of 0.76 J cm^-2^, cellular functions remained at different levels (Berney et al., 2006).

The capability to synthesize ATP after PL treatment (0.1–1.0 J cm^-2^) was examined with *E. coli* DSM 498, *L. innocua* DSM 20649, *Staphylococcus aureus* DSM 346 and *Salmonella enterica* ATCC BAA-1045. These bacteria still could generate ATP at different levels in a dose dependent manner. However, *L. innocua* and *E. coli* proved to be more resistant to PL (Kramer et al., 2017). Residual cellular activity was also measured through membrane potential, esterase activity, glucose uptake and pump activity (Kramer et al., 2016). The VBNC state was shown for *S. typhi* after PL treatment (Ben-Said et al., 2012).

Hilton et al. (2017) tested a potential synergistic effect between environmental temperatures (5–40°C) and PL. The results indicate that among tested bacterial strains, only *L. innocua* was slightly more inactivated at process temperatures of 40°C.

Tolerance and resistance development after PL application resulted in different outcomes. Heinrich et al. (2016a) described tolerance behavior of *L. monocytogenes* strains after homologous (multiple PL circles) and heterologous (combination with heat) stress application. *Ps. aeruginosa* and *E. faecalis* also seem to adapt to PL stress at low energy doses (Massier et al., 2012, 2013). Sublethal stress factors simulating technological hurdles (e.g., heat, salt, acid) may result in variable susceptibility. These aspects need more detailed evaluation. Also, a combination with other mild treatments, such as PEF and thermosonication was shown to improve PL efficiency (Kramer et al., 2016).

### Cold Plasma

Cold plasma (CP) treatment is a promising tool for the decontamination of food surfaces. However, the assessment of the plasma process is difficult because there is a lack of standardization of the process (use of different plasma sources, working gases, process parameters, etc.) and for each product a new assessment has to be conducted (Schlüter et al., 2013). Nevertheless, the following chapter gives an overview on CP treatment of foods, the underlying mechanisms, as well as the role of the VBNC state.

#### Principle of the Technology

In general, options for taking advantage of the so called fourth state of matter are manifold and widely used in various industries, e.g., illumination, material design, medicine, etc. However, when compared to the other non-thermal processes discussed in this chapter, cold atmospheric pressure plasma (CAPP) is a technique not industrially used for direct food treatment, yet.

Plasma is an ionized gas and can be generated in different ways. Basically, a process gas is forced to pass through an electric field. At a certain energy input [e.g., electron mean energy > 5 eV for air (Whitehead, 2016)] an ionizing process occurs at atmospheric pressure resulting in free electrons accelerated in the electric field. These free electrons can colloid with gas atoms or molecules resulting in an energy transfer and the generation of highly reactive species. These reactive species can then interact with food surfaces (Schlüter et al., 2013). Sources for the generation of CAPP are commonly plasma jets, corona discharges, dielectric barrier discharges (DBDs), and microwave discharges (Ehlbeck et al., 2011; Surowsky et al., 2015). The application of plasmas to food can be direct, semi-direct and indirect. In case the temperature does not exceed a value of 70°C the term CP was defined according to the blanching temperature as mild heat treatment (Schlüter et al., 2013). However, to treat heat-sensitive foods the maximum treatment temperature is often kept below 40°C.

In principle the treatment can be generated and applied batch wise (e.g., in a vacuum system), semi batch wise (e.g., in package when the electrodes are attached to the packaging material) or continuously for example in a bath of plasma processed water (PPW).

#### Inactivation and Mechanisms

**FIGURE 3:**
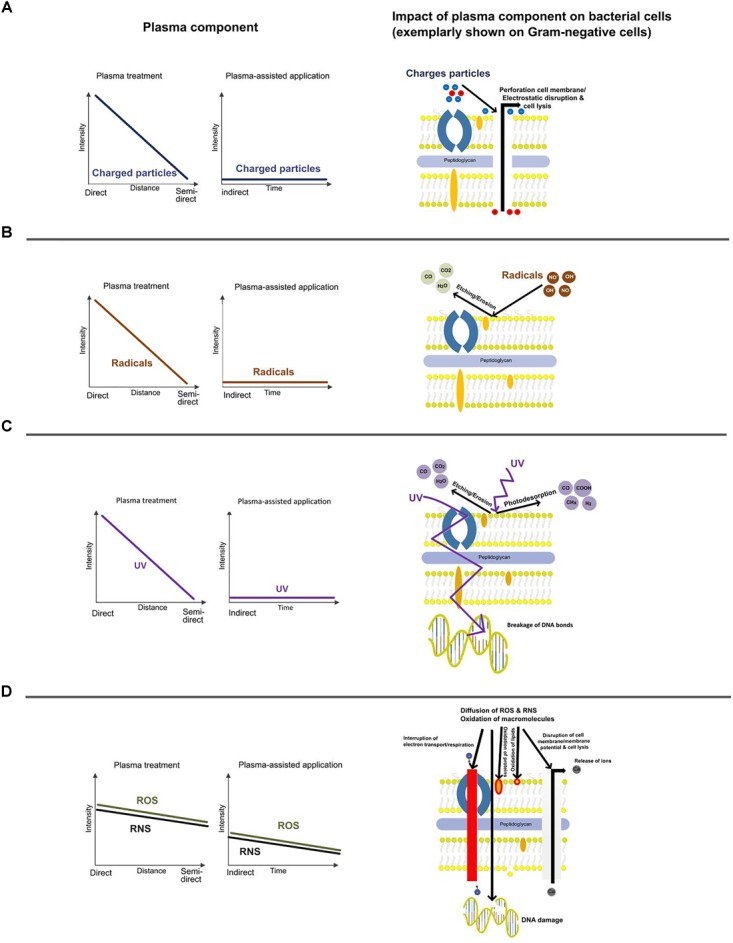
Schematic drawing of the main plasma effects on bacteria and their intensities with respect to the mode of operation. Impact of ions **(A)**, radicals **(B)**, UV-light **(C)**, and ROS/RNS **(D)** on Gram-negative bacteria.

The plasma-based inactivation of microorganisms is dependent on the mode of plasma application and matrix related effects. The effects of plasma on different matrices is described elsewhere (Surowsky et al., 2016). However, the plasma source and the mode of application strongly define the category of main species [e.g., ions, radicals, photons, ROS, reactive nitrogen species (RNS), etc.] and related effects (e.g., perforation, disruption, etching, photo desorption, diffusion, oxidation) (Figure 3). This is also of legal relevance since UV irradiation for example might result in a specific labeling of the treated food product.

There are various names and definitions for plasma applications available, but often the impact of the selected system on the main inactivation effect remains unclear. In Figure 3, the main aspects are categorized with respect to different set-ups of plasma application in food processing: direct, semi-direct, and indirect. Contact of the target organism with all sorts of species, including short-living species is just possible in the direct mode of action at shortest distances to the exited plasma. Semi-direct mode of operation means contact with selected reactive species. Depending on the system, contact with long living species and/or UV photons is intended. In a former description indirect treatment was defined as treatment with UV and/or VUV light and for plasma treatment of gases and liquids (Schlüter et al., 2013). Since PPW is a new option for fresh produce treatment on pilot scale (Andrasch et al., 2017), the term “indirect” must be re-defined here. More consistent “indirect” means that direct UV light is excluded and plasma species are suspended or dissolved in a transmitting media (e.g., PPW) and the PPW is subsequently used for a treatment. Since UV photons are part of the reactive species also UV lamps belong to the term “semi-direct.”

Since plasma interacts with the cell surface in the first instance, the properties of the cell envelope are a key aspect. Gram-negative bacteria contain a cell wall composed of two membranes: an outer membrane and an inner, cytoplasmic membrane. Compared to Gram-positive bacteria, only one layer of murein is present, located between the two membranes. Furthermore, lipopolysaccharides are attached to the outer membrane, acting as endotoxins after the destruction of the cell. Lipoproteins connect the outer membrane and the murein layer with each other, and although the outer membrane shows a low permeability, it contains pores (porins) which regulate the influx into the cell.

According to Schlüter and Fröhling (2014), perforation of microbial cell membranes, similar to the effect of PEF, can be the result of a plasma treatment. If the total tensile force of the membrane is exceeded by the total electric force an electrostatic disruption of the membrane occurs. The electric force is the result of a concentration of surface charge and in case of surface irregularities the electric force is even raised (Figure 3A).

Plasma treatment can result in erosion and etching induced by radicals attack (OH^⋅^ or NO^⋅^) of the cell membrane. The formation of volatile component (etching) is caused by absorption of radicals into the bacterial surface. Oxygen atoms or radicals emitted from the plasma are slowly combusted leading to an erosion of the microorganisms, atom by atom, through etching (Figure 3B).

UV light can induce intrinsic photodesorption which causes a breakage of chemical bonds in microorganisms and then the development of volatile by-products, such as CO, COOH, and CHx, from intrinsic atoms of the microorganisms. Dependent on the source and distance, UV-irradiation might lead to a destruction of genetic material (Figure 3C).

It must be considered that ROS react with cellular macromolecules, too. The cell envelope of Gram-negative bacteria consists of a thin layer of peptidoglycan and lipopolysaccharide, which is the major target for ROS. The inactivation is therefore mainly caused by cell leakage, accompanied by some DNA damage (Figure 3D). According to Han et al. (2016), the thick, rigid layer of peptidoglycan in the cell wall of Gram-positive bacteria remains intact upon ROS attack, leading mainly to inactivation due to intracellular damage such as DNA breakage.

However, the intensity of the described inactivation effects can be influenced by the mode of operation. The intensity generally decreases from direct to semi-direct treatment, but the effect of long-living ROS/RNS is then more pronounced and assumingly dominating the bacteria inactivation during plasma-assisted applications.

#### Role of Sublethal Injury and the VBNC State

Physical stresses like low or high temperatures, drying, irradiation, oxidative stress, starvation, PEFs, PL, and high pressure carbon dioxide or chemical disinfectants are known to induce bacteria to enter the VBNC state (Zhao et al., 2017) or bacteria are sublethally damaged (Silva et al., 2012). It is assumed that CP treatment also induces the VBNC state in bacteria which has to be taken into account during evaluation of inactivation efficiencies. Most studies dealing with the inactivation of bacteria by CP treatment do not consider uncultured bacteria (Brelles-Mariño, 2012). Food samples cannot generally be considered as free from pathogens after preservation treatments if plate count methods showed no colony growth because of the possible presence of VBNC or sublethally damaged bacteria. Besides the culturability of bacteria, detection of sublethally injured populations as well as the induction of the VBNC state after plasma application has to be evaluated to obtain a reliable assessment of the treatment. In case of laboratory experiments using pure cultures and non-selective media at least injured bacteria might also be detected. However, the inactivation of bacteria on food samples requires a specific detection of target microorganisms. Therefore the application of selective medium to suppress the background flora is necessary, which might lead to an underestimation of injured bacteria (Blackburn and McCarthy, 2000).

The treatment of *E. coli* in liquids by an atmospheric pressure plasma jet led to 7 log reduction according to plate count methods. In contrast, using the LIVE/DEAD Baclight Viability Kit only a 1 log reduction of *E. coli* was observed. Dolezalova and Lukes (2015) assumed that *E. coli* entered the VBNC state after plasma treatment but they were not able to resuscitate *E. coli* in nutrient broth even though they detected metabolic activity by mRNA analysis. Additionally, they observed plasma-induced DNA damages which hindered the replication and therefore the resuscitation was not successful. Thus, they proposed that the cells were in an active-but-non-culturable state and still able to be virulent because the cells still remained intact 3 months after plasma treatment. However, the measurement of membrane integrity is only one viability criterion and further analysis of metabolic activity would have been necessary to prove that the cells were really in an active-but-non-culturable state. The induction of the VBNC state by plasma treatment was also observed for *Bacillus stratosphericus* (Cooper et al., 2009). Cells of *B. stratosphericus* were treated with a DBD plasma either in suspension or surface-dried. Depending on the plasma dose applied, different viability states were obtained. With increasing plasma doses the viability status of *B. stratosphericus* shifted from viable and culturable to VBNC or disintegrated bacteria. Directly after plasma treatment the respiratory level of the bacteria cells was very low but 24 h later the respiratory activity increased eightfold. This implies the importance to evaluate the directly obtained inactivation but also the long-term inactivation efficacy. A dose-depending inactivation was also observed for *Chromobacterium violaceum* biofilms treated with an atmospheric pressure plasma jet (Joaquin et al., 2009). After 5 min exposure time almost all culturable biofilm cells were inactivated while a higher respiration rate was determined and even after 60 min of exposure residual metabolic activity was measured. It was proposed that the cells enter the VBNC state after 5 min of plasma treatment. However, no further analyses of the presumably VBNC cells were conducted to verify this assumption and to exclude the occurrence of sublethal damage instead of the VBNC state. Another study using an atmospheric pressure plasma jet to inactivate *Pseudomonas aeruginosa* biofilms also revealed the loss of culturability of the biofilm cells while showing intact cell membranes using the LIVE/DEAD viability kit (Mai-Prochnow et al., 2015). A presumably VBNC state after plasma treatment was also shown for *E. coli* biofilms (Ziuzina et al., 2015). Metabolic activity of *E. coli* biofilm cells was still measured after 300 s of DBD plasma treatment whereas the culturability was already lost after 60 s. Moreover, a rapid inactivation of planktonic methicillin-resistant *Staphylococcus aureus* (MRSA), *P. aeruginosa*, and *C. albicans* cells due to surface damage occurred after DBD plasma treatment (Kvam et al., 2012). With increasing treatment time membrane integrity was decreased, followed by leakage of intracellular components and finally a complete dissolution of the cell. The authors concluded that longer exposure times can avoid the induction of the VBNC state. However, all of the studies have in common that no further analyses of the presumably VBNC cells were conducted. Sublethal injury of bacterial cells as a result of plasma treatment is described in various studies (Rowan et al., 2008; Fröhling et al., 2012; Surowsky et al., 2014; Fröhling and Schlüter, 2015; Smet et al., 2016, 2017; Liao et al., 2017; Vaze et al., 2017; Dasan et al., 2018).

## Detection of Physiological and Structural Changes

In order to detect the individual physiological state of microbial populations and subpopulations after a novel decontamination treatment, a variety of different methodological tools is available. The selection of the appropriate method of analysis depends upon several factors, including the cellular target of the individual treatment, ease of use, and costs of investment for particular equipment, amongst others.

As injured cells are difficult to detect with traditional microbiological methods, culturing on conventional growth media is usually not possible (Kimura et al., 2017). Sublethally injured bacteria are typically analyzed by differential enumeration using non-selective and selective growth media (Espina et al., 2016). Alternative methods for the detection of bacterial viability are based on the measurement of cellular integrity, e.g., membrane potential and integrity, metabolic activity, e.g., DVC, detection of respiration, and detection of mRNA synthesis, or presence of nucleic acids, e.g., polymerase chain reaction (PCR), hybridization, cytochemical staining, reverse transcriptase PCR, nucleic acid sequenced-based amplification, strand displacement amplification; propidium monoazide (PMA)-PCR (viability PCR), loop-mediated isothermal amplification (LAMB) (Keer and Birch, 2003; Li et al., 2017). More specifically, applied methods for the detection of VBNC cells are acridine orange direct count (AODC), DVC, direct fluorescent antibody methods, p-iodonitrotetrazolium violet (INT) viability staining, 5-cyano-2,3-ditolyl tetrazolium chloride (CTC) staining, LIVE/DEAD^TM^ BacLight^TM^ Bacterial Viability Kit, microautoradiography, laser scanning cytometer-scanRDI, and flow cytometry (Babu et al., 2014), as well as Alexa Fluor^TM^ hydrazide (AFH) assay (Emerson et al., 2017), among others. The most relevant of these methods are explained in more detail in the following. Beforehand, some details on resuscitation from the VBNC state will be given, as basis for the afterward described analytical techniques.

### Resuscitation From the VBNC State

When kept at favorable conditions, microbial survivors, which underwent physiological stresses, e.g., during a decontamination treatment, and therefore developed sublethal injury or the VBNC state, can regain the ability to form colonies on growth media and therefore become fully vital again (Oliver, 2005; Ramamurthy et al., 2014). This process is called resuscitation and involves a variety of different metabolic pathways, gene expression, and repair mechanisms (Ayrapetyan and Oliver, 2016; Kimura et al., 2017). A crucial factor in this process, however, is the so-called resuscitation-promoting factor (Rpf), a cytokine (Mukamolova et al., 2002) present in bacteria able to enter the VBNC state, independent of their Gram behavior (Ramamurthy et al., 2014). Considering colony formation on microbiological growth media, the addition of agents able to convert ROS, e.g., catalase or sodium pyruvate, can help to reduce the content of said compounds, which can evolve during autoclaving of the media (Kong et al., 2004). Moreover, it was shown that if agar is replaced by other media, growth can be promoted again. This is associated with the occurrence of furan derivates, i.e., so-called neoformed contaminants that form during heat treatment (Ramamurthy et al., 2014). In some cases, an inversion of the stress responsible for the occurrence of the VBNC state can induce resuscitation, whereas other cultures require specific matrices and growth conditions (Ayrapetyan and Oliver, 2016). An overview of different pathogenic bacterial species known to enter the VBNC state and the corresponding resuscitation conditions is given by Li et al. (2014).

### Advanced Culture-Based Methods

The fact that injured cells cannot form colonies on selective media, whereas non-selective media enable the recovery and regrowth of the organisms, can be used to differentiate individual physiological states (Kell et al., 1998). For an effective recovery and detection of sublethally damaged bacteria the addition of sodium pyruvate, 3,3′-thiodipropionic acid, catalase, superoxide dismutase, Tween 80 or oxyrase is recommended to overcome possible toxic effects of ROS that are present in the growth media (Wesche et al., 2009; Wu, 2014). Liquid repair methods for the recovery of injured cells include the incubation of the sample in non-selective media for 1–5 h at 25–37°C, followed by subsequent enumeration by direct plating or most probable number techniques. Unfortunately, it is possible that non-injured and non-target cells multiply before the target cells are repaired. Even though the damaged cells can grow on non-selective media, the application for mixed cultures is hampered since the target cells cannot be differentiated (Wu, 2014). A more direct method for the differential detection of injured bacteria are solid-repair methods, e.g., pour-overlay plating, surface overlay plating, thin-agar-layer, agar underlay, as well as membrane or solid support-based methods. These methods combine non-selective agar media for recovery with the specific detection of target cells using selective media (Wu, 2008). However, high temperatures of the overlay methods may further affect injured cells (Wu, 2014). Additionally, the possible occurrence of enduring lag phases and vulnerability toward growth conditions exacerbate the detection of stressed or injured microorganisms (Joux and Lebaron, 2000; Hewitt and Nebe-von-Caron, 2004), and VBNC cells cannot be detected by culture-based methods (Colwell, 2009).

### Staining

**FIGURE 4:**
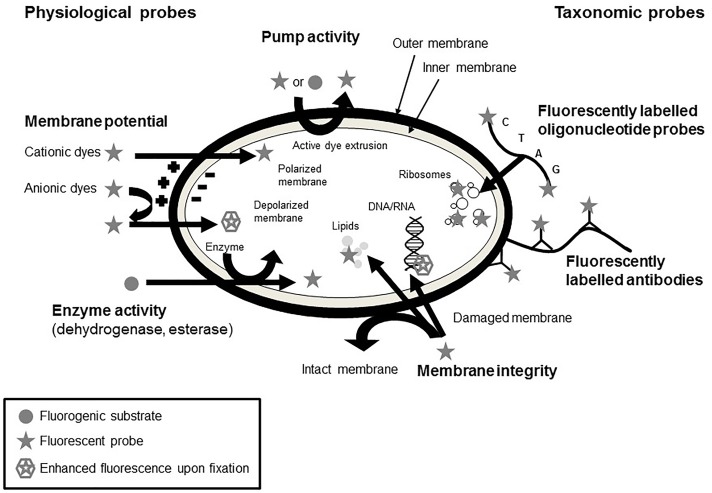
Mechanisms of action of different staining procedures for the evaluation of physiological fitness of microorganisms (adapted from Joux and Lebaron, 2000; Ben Amor, 2004).

A common methodology to visualize microbial physiology and viability is based on staining of cells using different functional dyes (see Figure 4). In general, these dyes possess certain properties, which allow them to enter cells or bind to certain regions inside the cell or to be metabolized. The occurrence of fluorescence then allows to draw conclusions on the binding or alteration of these dyes and consequently on the physiology of the cells. Common targets include the cell membrane, metabolic and enzyme activity, DNA, and the intracellular pH (Ueckert et al., 1995; Nebe-von-Caron et al., 2000). The prevailing staining methods are compiled hereafter.

The most commonly used method for the determination of bacterial viability is the measurement of membrane integrity using the LIVE/DEAD^TM^ BacLight^TM^ Bacterial Viability Kit. This method is based on the fact that dead cells are considered to have disrupted and/or broken membranes whereas viable cells are intact (Stiefel et al., 2015). The LIVE/DEAD^TM^ viability kit includes the membrane impermeant propidium iodide and the membrane permeant SYTO 9. A double staining with both dyes allows the discrimination of cells with compromised cell membranes from those with intact cell membranes. Evaluation can consequently be carried out microscopically and by flow cytometry (Boulos et al., 1999; Berney et al., 2007).

There are different fluorescent stains available to measure metabolic activity of bacteria. Alexa Fluor^TM^ hydrazide (AFH), a photostable fluorescent molecule, penetrates membrane-compromised cells and binds to carbonyl groups in irreversibly damaged proteins of dead, dying, and aging cells. The AFH assay is used for the detection of respiring bacteria in food and environmental samples. A double-staining with SYBR Green or DiOC_2_(3) showed that the majority of the dead cells would not be detected with DNA-binding dyes alone (Emerson et al., 2017).

The respiration activity of bacterial cells is often measured using CTC. In respiring cells the CTC is reduced to a red-fluorescent formazan molecule and accumulated within the cells. The reduced formazan can be detected and quantified in low concentrations. However, the detection by flow cytometry is more accurate than by epifluorescence microscopy (Sieracki et al., 1999). The INT assay also measures the activity of the electron transport system. INT is reduced to formazin in metabolically active cells and the formation and accumulation of formazin in the cells is an indicator for an active electron transport system (Li et al., 2014). However, studies on heat stressed cells showed an overestimation of viable *L. monocytogenes* using INT whereas CTC lead to an underestimation of viable cells but after a period of resuscitation it proved to be a good indicator for injured cells (Bovill et al., 1994).

The membrane potential of bacteria is involved in the glucose transport, chemotaxis, the generation of adenosine triphosphate (ATP), bacterial autolysis, and survival at low pH and is therefore an crucial part of bacterial metabolism (Novo et al., 1999). Membrane potential measurements are often conducted using merocyanine dyes, cyanine dyes, and oxonol dyes (Waggoner, 1979). Anionic lipophilic dyes, e.g., oxonol dyes, concentrate within cells and bind to lipid-rich components in case of a decreased membrane potential of the cells. The use of bis-(1,3-dibutylbarbituric acid)trimethine oxonol [DiBAC_4_(3)] to measure the membrane potential of Gram-negative and Gram-positive bacteria requires a pretreatment with ethylenediaminetetraacetic acid (EDTA). However, it has to be taken into account that the uptake of oxonol dyes is more dependent on membrane integrity than on the depolarization of the membrane (Joux and Lebaron, 2000). In contrast, cationic lipophilic cyanine dyes are able to easily enter into cells if they have a negative interior membrane potential gradient. Upon depolarization of the cells the dyes are released and upon hyperpolarization more dyes can enter the cells (Shapiro, 1994; Novo et al., 2000). Diethyloxacarbocyanine iodide [DiOC_2_(3)] allows to estimated membrane potential by radiometric techniques precisely. When excited at 488 nm, DiOC_2_(3) emits at 530 nm with an additional peak at <600 nm upon use of DiOC_2_(3) in elevated concentrations. The green fluorescence signal is not influenced by the membrane potential, whereas the red fluorescence signal is changing with an alteration in the membrane potential. The calculation of the ratio of the red to green fluorescence signal allows the determination of the membrane potential independently of the cell size (Novo et al., 1999). Another possibility to determine the maintenance of membrane potential is the utilization of the dye 2-NBDG ((2-[*N*-(7-nitrobenz-2-oxa-1,3-diazol-4-yl)amino]-2-deoxy-D-glucose). As this fluorescent molecule shows distinct analogies to glucose, it can enter the cells via the phosphoenol pyruvate phosphotransferase system (Yoshioka et al., 1996; Natarajan and Srienc, 1999). After uptake of 2-NBDG, a green fluorescence is emitted, which is gradually decreased as the molecule is degraded by the cell’s metabolism (Yoshioka et al., 1996). In cells with a reduced viability, this glucose uptake cannot take place anymore and thus indicates the loss of the membrane potential (Berney et al., 2006).

Cellular viability, in terms of enzyme activity, more specifically esterase activity, is another parameter that is measured by flow cytometry. Lipophilic, uncharged and non-fluorescent fluorogenic substrates are generated by esterification of polar, membrane permeable fluorescent dyes with non-fluorescent acetyl or acetoxymethyl esters. The non-fluorescent dye enters cells and is then hydrolyzed by esterases resulting in a fluorescent product which remains in cells with intact cell membranes. Hydrolyses of fluorescein diacetate to fluorescein leads only to low fluorescent signals and retention within the cells is limited. In contrast, carboxyfluorescein diacetate (cFDA), chloromethylfluorescein diacetate (CMFDA), and carboxyfluorescein diacetate acetoxy methyl ester (cFDA-AM) are hydrolyzed into hydrophilic products and therefore, the retention within the cells is more pronounced (Ueckert et al., 1995; Joux and Lebaron, 2000; Veal et al., 2000).

For the microautoradiographic method the sample is exposed to radioactive isotypes and the labeled radioactive isotope can be located within the cells. The combination of radiography with fluorescence *in situ* hybridization (FISH) allows the direct detection of viable cells in complex environments (Babu et al., 2014). The laser scanning cytometer, scanRDI, is a solid-phase cytometry technique where a fluorescent stain is used to evaluate the metabolic activity and membrane integrity of viable cells by scanning the complete membrane. This allows the detection of a low number of fluorescently labeled cells (Babu et al., 2014).

A comprehensive overview on fluorescent dyes for the assessment of microbial physiology as well as detailed mechanisms of action are given by Joux and Lebaron (2000).

### Direct Viable Count (DVC)

Using DVC, viable cells can be identified microscopically. The samples are incubated after addition of nutrients and a specific antibiotic (nalidixic acid), which is capable of inhibiting DNA gyrase (Kogure et al., 1979). The cell division is inhibited but available nutrients are metabolized, resulting in the elongation of the cells. These cells are considered as viable, whereas non-elongated cells are considered to be metabolically inactive (Buchrieser and Kaspar, 1993; Besnard et al., 2000a). Unfortunately, Gram-positive species are resistant to nalidixic acid and therefore not inhibited so that this method is not reliable. However, the combination of DVC with FISH can enhance the reliability of the method (Babu et al., 2014). The substitution of nalidixic acid with another antibiotic, ciprofloxacin, allows the application for Gram-negative as well as Gram-positive bacteria (Buchrieser and Kaspar, 1993). AODC is a differential staining method, in which acridine orange (AO) interacts with RNA to red-orange fluorescent and with DNA to green fluorescence complexes. This allows the differentiation of viable (red) from dead (green) cells. However, the concentration of AO, the pH of the growth medium, and the growth medium used as well as the incubation time lead to a high variance in fluorescence color and hampers the clear classification of injured, dead and viable cells (Babu et al., 2014). A further possibility is double staining of the cells using CTC and 4′,6-diamidino-2-phenylindole (DAPI). In this method, total counts emit red and blue fluorescence, whereas the viable cells can be distinguished by their red color. The blue staining is due to DAPI, which colors all cells, the CTC, on the other hand, is reduced by the active cells and consequently shows red fluorescence (Rodriguez et al., 1992; Besnard et al., 2000b).

The technique can be used to analyze microorganisms in a variety of different foods and biological media (Comas-Riu and Rius, 2009), such as dairy (Pettipher et al., 1983; Rowe and McCann, 1990) and meat (Shaw et al., 1987; Duffy and Sheridan, 1998) products, as well as alcoholic beverages, including wine (Divol and Lonvaud-Funel, 2005) and beer (Siegrist et al., 2015). Thus, DVC is a fast and versatile method for the analysis of the physiological status of microbial cells. However, several sources of interference exist, i.e., stains can unspecifically start reactions with compounds of carbon present in the matrix, and interact with preservatives, such as sorbic acid (Comas-Riu and Rius, 2009).

### Molecular Biological Methods

As live/dead staining using microscopy or flow cytometry is unspecific, an identification of the stained bacteria is not possible. Therefore, the combination of PCR with viability dyes allows the monitoring of decontamination efficiencies in environmental samples and of a product’s natural microflora (Elizaquível et al., 2014). Viability PCR is commonly performed using ethidium bromide monoazide (EMA) or propidium iodide monoazide (PMA). The samples are stained with EMA or PMA, which can enter perforated cell membranes and bind to DNA. The photooxidation at 464 nm leads to irreversible damage of the nucleic acids. In consequence, these cells cannot be amplified and only cells with intact membranes are amplified (Trevors, 2012). The analysis of samples with and without PMA allows the evaluation of live and dead ratios of a microbial population (Emerson et al., 2017). However, there are some drawbacks of these methods. Cells can remain intact but do not show metabolic activity resulting in false-positive detection of viable cells. Additionally, viable species can have perforated cell membranes during growth, cell wall synthesis or during injury resulting in false-positive detection of dead cells (Stiefel et al., 2015).

Flow cytometry, as a method to continuously analyze a great amount of individual microorganisms within a population, is widely used due to its rapid and versatile detection abilities. By isolating cells and individually passing them through a laser beam, the particle size can be determined by detection of scattering (Comas-Riu and Rius, 2009). Furthermore, the use of different fluorescent dyes for viable staining (see the Section “Staining”) allows the evaluation of individual physiological states and cellular targets of antimicrobial treatments, among others (Li et al., 2014; Ayrapetyan and Oliver, 2016). This includes membrane intactness and potential, activity of efflux pumps, as well as activity of several enzymes. By using taxonomic probes, e.g., fluorescently labeled oligonucleotides or antibodies, it is further possible to taxonomically classify the analyzed microbial cultures (Joux and Lebaron, 2000).

Moreover, several assays exist to detect the physiological status of microorganisms. Among them is the deoxyribonuclease (DNase) I protection assay, which indicates the level of membrane integrity, as only perforated membranes admit the intrusion of the enzyme and the associated degradation of genomic DNA. It has already successfully been implemented for the determination of the VBNC state in *Yersinia pestis* (Pawlowski et al., 2011). Using the p-iodonitrotetrazolium violet (INT) assay, metabolic activity can be evaluated (see the Section “Staining”). This assay can thus be used to detect the VBNC state, e.g., as shown by Rahman et al. (1994) for *Shigella dysenteriae* Type 1. Another assay, BacTiter-Glo^TM^, uses luciferase to detect ATP concentration as an indication of metabolically active organisms. Exemplarily, it could contribute to detect the VBNC state in *L. monocytogenes* (Lindbäck et al., 2010).

## Further Research Needs

A successful implementation of novel preservation technologies in the production chain requires a detailed knowledge of inactivation effects and recovery phenomena in order to derive necessary treatment parameters and concepts. To ensure product safety, inactivation data cannot only rely on colony count monitoring immediately after the treatment but have to be verified during and at the end of the shelf life. In addition, a variety of cellular targets needs to be investigated in order to comprehensively describe the physiological and structural state of a microbial cell. For this purpose, online analytical tools are required. Flow cytometry is a promising tool and miniaturized versions are in development to be implemented before and after the inactivation step in order to gain information on cellular damage as well as the recovery potential. An online-flow cytometric measurement firstly requires an optimization of the staining methods as well as an improvement of the analysis and interpretation of flow cytometric data to allow a reliable application of prediction models to assess the impact of decontamination treatments on bacterial physiology. Additionally, the development of new fluorescent dyes that allow the simultaneously detection of physiological and structural properties of bacteria would improve the detection of the viability status of bacteria after inactivation treatments.

In addition, selective inactivation and colony count reduction by different mechanisms and its implication on the development of the microbial population and selective recovery, growth and adaptation of microorganisms needs to be studied. In this context, the microbial diversity changes as result of inactivation treatments should also be considered to avoid potential (re)growth of human pathogenic bacteria due to the lacking presence of naturally occurring phyllospheric and endophytic bacteria after inactivation treatments.

## Conclusion

In summary, it can be stated that both the VBNC state as well as sublethal injuries can pose significant threats to food safety, as an underestimation of the microbiological status after a decontamination treatment can occur. Under favorable conditions, microbial subpopulations might be able to overcome this inactive state and become active again, thus being able to reproduce within the respective matrix. Hence, recovery phenomena need to be taken into account when evaluating the inactivation effectiveness in particular in the case of novel technologies. In order to avoid the necessity to apply severe treatment intensities with sufficient safety margins but overprocessing and quality losses at the same time, it is crucial to not only understand inactivation of microbial cells but also their recovery strategies and to develop suitable processing schemes, e.g., based on the hurdle concept. Otherwise, an increased spoilage can occur, leading to an unacceptable product quality loss already prior to reaching the “best before” date. More importantly, the presence of pathogenic species in food may cause a serious outbreak of certain foodborne diseases. Hence, cellular targets of novel preservation technologies such as HHP, PEF, PL, and CP and related inactivation and recovery mechanisms need to be studied in order to design safe processes with minimized alteration of product quality.

This also underlines the importance of specific and accurate analytical tools being able to distinguish between the individual states of microbiological subpopulations, i.e., alive, VBNC/sublethal injury, and dead species. The presented methodologies differ in accuracy and in rapidity of the detection, as well as in the individual level of sophistication, costs of analysis, and ease of use. Depending on the method, a false-positive or false-negative detection of certain species might occur. Exemplarily, this can be the case if intact cells appear to possess no more metabolic activity (false-positive) or due to naturally occurring phenomena, such as the occurrence of pores in the cell membrane during certain growth stages (false-negative). This, however, may lead to an over or underestimation of the food safety status of the analyzed product. Therefore, it is recommended that several viability indicators (e.g., culturability, metabolic activity, membrane integrity) should be measured for a reliable detection of viable cells (Elizaquível et al., 2014).

## Author Contributions

MZ-P contributed to the pulsed light section. AK and HJ contributed to HPP and PEF section. OS contributed to plasma section. FS and AF equally contributed to the manuscript as co-first authors.

## Conflict of Interest Statement

The authors declare that the research was conducted in the absence of any commercial or financial relationships that could be construed as a potential conflict of interest. The reviewer GM and handling Editor declared their shared affiliation.
